# Associations between physical activity, sedentary behaviour, and alcohol consumption among UK adults: Findings from the Health Behaviours during the COVID-19 pandemic (HEBECO) study

**DOI:** 10.1371/journal.pone.0287199

**Published:** 2023-10-10

**Authors:** Lady Gwendoline Akwa, Lesley Smith, Maureen Twiddy, Grant Abt, Claire Garnett, Melissa Oldham, Lion Shahab, Aleksandra Herbec

**Affiliations:** 1 Faculty of Health Sciences, Institute of Clinical and Applied Health Research, University of Hull, Hull, United Kingdom; 2 Hull York Medical School, University of Hull and University of York, Hull, United Kingdom; 3 Department of Sport, Health and Exercise Science, University of Hull, Hull, United Kingdom; 4 Department of Behavioural Science and Health, UCL, London, United Kingdom; 5 Centre for Behaviour Change, UCL, London, United Kingdom; 6 Institute-European Observatory of Health Inequalities, Calisia University, Kalisz, Poland; Universitas Mercatorum, University of the System of the Italian Chambers of Commerce, ITALY

## Abstract

**Introduction:**

The COVID-19 pandemic and attendant lockdowns have had a substantial negative effect on alcohol consumption and physical activity globally. Pre-pandemic evidence in the adult population suggests that higher levels of physical activity were associated with higher levels of drinking, but it is unclear how the pandemic may have affected this. Therefore, this study aims to assess the association between alcohol consumption and physical activity in a UK cohort established during the COVID-19 pandemic.

**Methods:**

Analyses utilized data from the Health Behaviours during the COVID-19 pandemic (HEBECO) study involving 2,057 UK adults (≥18 years). Participants completed self-report measures of alcohol consumption [frequency, quantity, frequency of heavy episodic drinking (HED) and AUDIT-C score] and physical activity [moderate-vigorous physical activity (MVPA), frequency of muscle strengthening activity (MSA) and sedentary behaviour] between November 2020 and January 2021. Ordinal logistic regression models were conducted, adjusting for sociodemographic factors.

**Results:**

Fifteen percent of the sample reported abstinence from drinking. Overall, 23.4% of participants drank ≥4 times/week, 13.9% drank more than 6 units/single drinking occasion (HED), 7.5% reported HED daily/almost daily and 4.2% scored ≥11 on AUDIT-C. MSA 3 days/week compared with no MSA was significantly associated with higher odds of alcohol frequency [OR (95 CI%) = 1.41 (1.04–1.91)], quantity [OR (95 CI%) = 1.38 (1.02–1.87)], HED [OR (95 CI%) = 1.42 (1.05–1.94)] and possible dependence [OR (95 CI%) = 1.47 (1.05–2.06)]. The association of MVPA and sedentary behaviour with drinking measures was not significant (p>0.05).

**Conclusion:**

In contrast with previous research, MSA rather than aerobic physical activity was associated with increased alcohol consumption during the COVID-19 pandemic. It is conceivable that during lockdown while drinking was used as a coping strategy, limited opportunities for aerobic exercise made MSA a more convenient form of physical activity. To guide public health interventions, more research is required to examine the temporal relationship between different forms of physical activity and alcohol consumption.

## Introduction

Alcohol consumption and physical inactivity are leading risk factors of disease and mortality globally [1]. Alcohol consumption accounts for an estimated 5.3% of all deaths [2] and physical inactivity accounts for 7.2% of deaths worldwide [3]. The 2016 UK Chief Medical Officers’ guidelines for low-risk drinking recommend that adults should not consume more than 14 units/week, spread out evenly over 3 or more days to prevent heavy episodic drinking (HED) (more than 6 units per drinking occasion). However, pre-pandemic estimates suggest that about 30% men and 15% women exceed this low-risk threshold [4]. Meanwhile about 27% of adults in the UK fail to meet at least 150 minutes per week of moderate to vigorous activity (MVPA) as recommended in the UK Chief Medical Officers’ physical activity guidelines and even fewer meet the recommendations for muscle strengthening activity (MSA) of at least twice a week [4].

Extensive evidence suggests that alcohol consumption and physical inactivity are independently associated with non-communicable diseases such as cancer and cardiovascular disease (CVD) [2,3]. Large prospective studies of adults in the UK found evidence that physically active individuals are at less risk of all-cause, CVD and cancer mortality associated with alcohol consumption [5,6]. It is therefore plausible that increasing physical activity levels may offset some risks associated with alcohol consumption, but biological mechanisms are not yet fully understood. In animal studies, alcohol-induced oxidative stress damage to the heart has been diminished with regular physical activity through its regulatory effect on antioxidant capacity, heart rate and blood pressure [7]. Alcohol consumption and physical activity may also act in similar but opposing pathways on hormones, lipid profiles and blood plasma in the development of some cancers [7,8].

In adults, high levels of physical activity have been associated with high levels of alcohol consumption [9–11]. This positive relationship has largely been reported among studies conducted in the US and among college students, with few studies on the general population and countries such as the UK. Only one study to our knowledge has investigated the association between physical activity and alcohol consumption in the general UK adult population. This study found a positive association between sports participation and heavy drinking and some of the association was accounted for by sports club membership [12]. However, this study only controlled for age and gender and did not examine the independent association of MVPA, MSA and sedentary behaviour with alcohol consumption. The possible mechanisms underlying this alcohol-physical activity relationship are unclear and complex. One hypothesis is based on the notion that ‘work hard, play hard’ is a motivating principle because individuals are motivated to put maximum effort in all rewarding activities [13]. Both alcohol and physical activity stimulate the mesocorticolimbic pathway of the brain, which is linked with biological reward processes [14]. Individuals may therefore engage in both physical activity and drinking to prolong positive affect. Further, individuals may be motivated to increase physical activity to mitigate risks of excessive drinking such as increased consumption of empty calories [13]. This potentially justifies the relevance of BMI as a possible covariate in the alcohol-physical activity association.

The Coronavirus (COVID-19) pandemic triggered a series of public health strategies such as social distancing and lockdowns to control the spread of infection in the UK. These measures resulted in extended periods of isolation, loneliness, and stress [15]. Among adults, there were reported increases in the prevalence of high risk drinking [16,17] and HED [18]. Physical activity also declined in the UK because individuals were allowed only one bout of exercise outside in a day while gyms and recreational facilities were closed [19]. Increased amounts of discretionary time during lockdowns also facilitated a rise in sedentary behaviour from time spent sitting to watch television or use smart devices such as phones or computers for either work or school [20]. It is probable that during lockdown changes in drinking patterns and physical activity levels may have been linked in a few ways. Physical activity and alcohol consumption may have been used to relieve stress and boredom from isolation [21,22]. It is also likely that PA could have been used to offset the harms of increased drinking, or increased consumption might have reduced physical activity due to hangovers. Different sociodemographic factors were independently associated with alcohol consumption and physical activity during lockdowns in the UK [16,23] but we do not know how these factors might influence the association between alcohol consumption and physical activity.

Understanding the association between PA and alcohol consumption is valuable for developing public health messages in potential future pandemics. As an example, messages pertaining to a potentially harmful to health association such as high alcohol intake associated with low physical activity, may underscore the potential ramifications of increased caloric intake coupled with decreased energy expenditure. Considering the scarcity of evidence on this association during the early and later phases of the COVID-19 pandemic in the UK, the timely and comprehensive data used in this study fills a critical gap. Therefore, we hypothesised that there is an:

1. Association of physical activity [total weekly moderate to vigorous aerobic physical activity (MVPA), frequency of muscle strengthening activity (MSA) and sedentary behaviour] with alcohol consumption [frequency of drinking, quantity of drinking, frequency of heavy episodic drinking (HED) and risky drinking status as defined by Alcohol Use Disorders Identification Test-Consumption (AUDIT-C) score].
2. Effect of smoking status and sociodemographic factors on the association between physical activity and alcohol consumption.

## Methods

### Study design

The Health Behaviours during the COVID-19 pandemic (HEBECO) study is a retrospective longitudinal online study examining self-reported health behaviours (alcohol, smoking, diet, and PA) of UK-based adults (aged 18 years and above) during the COVID-19 pandemic. The current study used cross-sectional data from the 6-month follow-up survey of HEBECO for analysis. The protocol for the present analysis was pre-registered on the Open Science Framework (https://osf.io/26q5f/). Departures from the statistical analysis plan are explained in below (Further analysis section). The reporting follows the Strengthening the Reporting of Observational Studies in Epidemiology guidelines (STROBE) [24].

The study was approved by the UCL Research Ethics Committee at the UCL Division of Psychology and Language Sciences (CEHP/2020/579) and the University of Hull Faculty of Health Sciences Research Ethics Committee (REF FHS421). Participants gave informed written consent before taking part in the study.

### Participants

HEBECO recruited a convenience sample of UK adults via paid and unpaid advertisements on social media, and email invitations across universities, charities, Public Health England, Cancer Research UK, and local authorities across the UK. In total, 2,992 adults participated in the HEBECO study at baseline between April and June 2020. The 6-month follow-up wave was conducted between November 2020 and January 2021, corresponding to the reintroduction of tighter restrictions and tiered lockdowns in various parts of the country. Full details of the timeline of UK COVID-19 lockdowns and measures are available [25].

### Measures

Alcohol consumption. The Alcohol Use Disorders Identification Test (AUDIT-C) was used to measure alcohol consumption [26]. Alcohol frequency was measured by the question, ‘How often did you have a drink containing alcohol in the past 6 months?’. Alcohol quantity was measured by the question, ‘In the past 6 months, how many units of alcohol did you drink on a typical day when you were drinking?’. Frequency of HED was assessed with the question, ‘In the past 6 months how often did you have 6 or more units on one occasion?’. All responses for alcohol frequency, quantity, and frequency of HED were categorised based on the AUDIT-C scale. Total AUDIT-C scores were categorised such that scores of 0–4 indicated ‘low risk’, 5–7 ‘increasing risk’, 8–10 ‘higher risk’ and >10 ‘possible dependence’. The AUDIT-C categories are classified as risky drinking status hereafter and were used as an indicator of possible dependence since the AUDIT-C is a screening tool and not a diagnostic tool.

Physical activity. Participants were asked to rate the frequency of aerobic activity per week and the duration per session in the past month using a validated physical activity tool [27,28]. Total MVPA was determined as a product of the frequency and duration (hrs/week). Participants reported the frequency of MSA (days/week) and were categorised as ‘0’, ‘1’, ‘2’, ‘3’ and ‘4 or more’ days/week. Examples of MSA included Pilates, push-ups, squats, yoga, and exercises involving free weights (dumbbells or alternatives), weight machines and elastic resistance bands. Average daily sedentary time was assessed by the average number of hours participants spent sitting (e.g., working, travelling, studying, watching TV etc) on weekends and weekdays (hours/day).

Socio-demographic information. Socio-demographic information included age (years), gender (participants were asked to select which they identify most with, and responses were dichotomised into males, which includes non-binary/prefer not to say, or females), ethnicity (white; other), educational status (post age 16; no post age 16 educational qualifications), and employment status (employed; other). Body mass index (BMI) was categorised as underweight and normal (<18.5–24.9kg/m^2^), overweight (25.0–29.9 kg/m^2^), obese (30.0->39.9 kg/m^2^) [29]. Smoking status was measured by the question, ‘Which of the following best applies to you now?’. Response options were a. ‘I smoke cigarettes (including hand-rolled) everyday,’ b. ‘I smoke cigarettes (including hand-rolled) but not every day,’ c. ‘I do not smoke cigarettes at all, but I do smoke tobacco of some kind (e.g., pipe, cigar or shisha), d. ‘I have stopped smoking or using tobacco completely in the past 3 months’, e. ‘I stopped smoking completely more than 3 months ago’ and f. ‘I have never been a regular smoker (i.e., smoked for a year or more)’. Participants who selected a, b and c were categorised as ‘current smoker’, those who selected d and e were categorised as ‘ex-smoker’ and ‘never smoker’ as those who selected f.

### Statistical analyses

All analyses were conducted in IBM SPSS Statistics Version 24 (SPSS, Inc. Chicago). Results are presented as means (standard deviation), frequencies (percentages), odds ratios and 95% confidence intervals (95% CI). Descriptive statistics were calculated to characterise participants’ demographics by gender. Ordinal logistic regression was conducted to examine separately the associations of each PA variable and each alcohol consumption variable. Unadjusted and adjusted models were examined. The level of significance was set at p<0.05 and the Benjamini-Hochberg procedure was used to correct for false discovery rate [30].

Sensitivity analysis. In sensitivity analysis, ordinal regression models were repeated with weighted data (98^th^ percentile) obtained from the Office for National Statistics to account for the non-random sample using the *weight cases* command in SPSS. Additionally, we conducted unplanned exploratory analysis to examine whether meeting PA guidelines can be associated with drinking more. The rationale for exploratory analysis was to provide real-world inferences with proven consequences on health and mortality. PA variables were dichotomised based on the UK Chief Medical Officers’ PA guidelines. Total MVPA was categorised as ‘<150mins/week’ and ‘≥150mins/week’ and frequency of MSA was categorised as ‘<2 days/week’ and ‘≥2 days/week’. Average daily sedentary time was categorised as ‘<7.5hrs/day’ and ‘≥7.5 hrs/day’ based on the dose response relationship between sedentary time and all-cause mortality [31]. Alcohol consumption variables were also dichotomised: frequency as ‘frequent’ and ‘never’; quantity as ‘<6 units/day’ and ‘≥6 units/day’ and frequency of HED as ‘no HED’ and ‘any HED’. Risky drinking status was also categorised as ‘low risk’ and ‘high risk drinking’. Separate binary logistic regression (adjusted and unadjusted) models were used to examine the association between these dichotomised variables.

## Results

Descriptive data of participants’ socio-demographic characteristics, alcohol consumption and PA are shown in Table 1. Out of the 1,944 participants in the 6-month follow-up wave, a total of 1,892 adults (weighted N = 1,703) had complete data and were included in the final analyses. Age ranged between 18 and 91 with a median age of 54 years. Participants were predominantly white (96%) and female (71%). Overall, 15% reported abstinence from drinking in the previous 6 months. On a typical drinking day, about 14% reported drinking more than 6 units and 4% engaged in HED daily/almost daily. More males than females engaged in HED daily/almost daily and were possibly alcohol dependent (>10 score on the AUDIT-C).

**Table 1:** Participant characteristics stratified by gender (weighted).

Characteristics	Females (N = 1346)	Males^a^ (N = 546)	Total (N = 1892)
Age (years), m(SD)	49.3 (15.3)	50.7 (16.3)	50.0 (15.8)
Rating of physical health, m(SD)	3.4 (1.1)	3.4 (1.0)	3.4 (1.0)
*Ethnicity*, *n (%)*			
White	781 (87.2)	746 (92.4)	1527 (89.7)
Other^b^	115 (12.8)	61 (7.6)	176 (10.3)
*Educational status*^*c*^, *n (%)*			
Post age 16	571 (63.7)	520 (64.4)	1091 (64.0)
No post age 16	326 (36.3)	287 (35.6)	613 (36.0)
*Employment status*, *n (%)*			
Employed	510 (56.9)	430 (53.3)	940 (55.2)
Other^d^	386 (43.1)	377 (46.7)	763 (44.8)
*BMI (kg/m*^*2*^*)*, *n (%)*			
Underweight/normal (<18.5–24.9kg/m^2^)	373 (41.6)	288 (35.7)	661 (38.8)
Overweight (25.0–29.9 kg/m^2^)	247 (27.6)	302 (37.4)	549 (32.2)
Obese (30.0->39.9 kg/m^2^)	200 (22.3)	168 (20.8)	368 (21.6)
Other^e^	76 (8.5)	49 (6.1)	125 (7.3)
*Smoking status*, *n (%)*			
Non-smoker	527 (58.8)	400 (49.6)	927 (54.4)
Ex-smoker	206 (23.0)	234 (29.0)	440 (25.8)
Current smoker	163 (18.2)	173 (21.4)	336 (19.7)
**Alcohol consumption**			
Current abstainers	204 (22.8)	115 (14.3)	319 (18.7)
*Frequency of alcohol consumption*, *n (%)*			
Monthly or less	175 (19.6)	115 (14.3)	290 (17.0)
2 to 4 times per month	193 (21.6)	152 (18.8)	345 (20.3)
2 to 3 times a week	187 (20.9)	239 (29.6)	426 (25.0)
4 or more times a week	136 (15.2)	186 (23.0)	322 (18.9)
*Quantity of alcohol consumed (units/drinking occasion)*, *n (%)*			
1 to 2	304 (33.9)	207 (25.7)	511 (30.0)
3 to 4	184 (20.5)	130 (16.1)	314 (18.4)
5 to 6	117 (13.0)	111 (13.8)	228 (13.4)
7 to 9	48 (5.4)	96 (11.9)	144 (8.5)
10 or more	40 (4.5)	148 (18.3)	188 (11.0)
*Frequency of heavy episodic drinking*, *n (%)*			
Never	349 (39.0)	189 (23.4)	538 (31.6
Monthly	141 (15.8)	152 (18.8)	293 (17.2)
Less than monthly	68 (7.6)	107 (13.3)	175 (10.3)
Weekly	104 (11.6)	190 (23.5)	294 (17.3)
Daily or almost daily	29 (3.2)	54 (6.7)	83 (4.9)
*Risky drinking status*, *n (%)*			
Low risk	609 (68.0)	376 (46.6)	985 (57.8)
Increasing risk	155 (17.3)	157 (19.5)	312 (18.3)
Higher risk	107 (11.9)	206 (25.5)	313 (18.4)
Possible dependence	25 (2.8)	68 (8.4)	93 (5.5)
**Physical activity**			
*Frequency of MSA (days per week)*, *n (%)*			
0	569 (63.5)	514 (63.8)	1083 (63.6)
1	112 (12.5)	81 (10.0)	193 (11.3)
2	78 (8.7)	48 (6.0)	126 (7.4)
3	69 (7.7)	62 (7.7)	131 (7.7)
4 or more	68 (7.6)	101 (12.5)	169 (9.9)
Total MVPA (hours per week), median (IQR)	1.5 (2.8)	1.5 (3.3)	1.5 (1.5)
Average daily sedentary time (hours per day), m(SD)	7.4 (3.6)	8.0 (3.7)	7.7 (3.6)

BMI = Body Mass Index; MSA = Muscle strengthening activities; MVPA = Moderate to vigorous physical activity.

^a^Includes non-binary and prefer not to say.

^b^Mixed/Multiple ethnic groups, Asian/ Asian British, Black/African/Caribbean/Black British, Chinese, Arab, other ethnic groups and prefer not to say.

^c^Post age 16 qualification includes A-level/Higher school certificate or equivalent (e.g. NVQ3), Bachelor’s degree or equivalent (e.g. NVQ4), Masters/PhD/PGCE or equivalent and other higher qualifications. No post age 16 qualification includes no formal qualification, GCSE/School Certificate/O-level/CSE and Vocational qualifications (e.g. NVQ1+2).

^d^Students, furloughed/laid off during COVID-19, retired, unemployed before COVID-19, homemakers/fulltime parents/carers, those unable to work due to disability and other.

^e^Prefer not to say/don’t know.

The average time spent in MVPA per week was 3.0 hours but there was large variation across participants (SD = 4.7). Out of the 61.5% who did not meet the recommended 150 minutes/week of MVPA, 19% reported not engaging in any MVPA. Almost three-quarters (71.1%) of participants did not meet the recommendations for muscle strengthening activity of at least two days a week, about 58% of which engaged in no muscle strengthening activities per week. Although males reported higher levels of total MVPA, females engaged in less sedentary activity, averaging 7.2 hours/day compared with 8.1 hours/day in men.

### Association between physical activity and alcohol consumption variables

Unadjusted and adjusted ordinal logistic regression models examining the association between PA and alcohol consumption variables are presented in Table 2. In unadjusted models, the odds of being in a higher category of any alcohol consumption measure was not significantly associated with higher levels of total MVPA (Table 2). However, the odds of being in a higher category of alcohol quantity, frequency of HED and risky drinking status increased by 5% with each unit increase in average daily sedentary time. Engaging in MSA 3 days per week compared to no MSA was associated with higher odds of drinking frequency, quantity, frequency of HED and risky drinking status.

**Table 2:** Association between physical activity and alcohol consumption variables (unadjusted and adjusted models).

Independent variables (reference category)	Frequency of alcohol consumption		Quantity of alcohol consumed (units)		Frequency of HED		Risky drinking status	
Unweighted N = 1892	OR (95% CI)	P	OR (95% CI)	P	OR (95% CI)	P	OR (95% CI)	P
***Unadjusted models***								
Total MVPA, *hrs/week*	1.01 (1.00–1.03)	0.169	1.01 (0.99–1.02)	0.541	1.01 (0.99–1.02)	0.488	1.00 (0.98–1.02)	0.800
Average daily sedentary time, *hrs/day*	1.00 (0.98–1.02)	0.971	1.05 (1.02–1.08)	<0.001*	1.05 (1.02–1.08)	<0.001*	1.05 (1.02–1.08)	<0.001*
Frequency of MSA, *days/week* (0 days/week)								
1	1.17 (0.92–1.49)	0.204	1.02 (0.80–1.30)	0.905	1.02 (0.80–1.31)	0.847	0.94 (0.72–1.23)	0.655
2	1.14 (0.87–1.49)	0.355	1.03 (0.79–1.36)	0.813	1.00 (0.76–1.31)	0.990	0.89 (0.65–1.20)	0.433
3	1.46 (1.08–1.99)	**0.013**	1.39 (1.03–1.88)	**0.030**	1.49 (1.10–2.01)	0.010*	1.40 (1.02–1.93)	**0.040**
4 or more	1.23 (0.93–1.62)	0.144	1.11 (0.84–1.46)	0.466	1.18 (0.89–1.56)	0.246	1.09 (0.81–1.48)	0.566
***Adjusted models*** ^*a*^								
Total MVPA, *hrs/week*	1.00 (0.99–1.02)	0.733	1.00 (0.98–1.02)	0.926	1.00 (0.98–1.02)	0.971	0.99 (0.97–1.01)	0.492
Average daily sedentary time, *hrs/day*	1.00 (0.98–1.03)	0.805	1.01 (0.99–1.04)	0.430	1.01 (0.98–1.04)	0.500	1.01 (0.99–1.04)	0.354
Frequency of MSA, *days/week* (0 days/week)								
1	1.15 (0.90–1.47)	0.275	0.99 (0.77–1.28)	0.947	0.99 (0.77–1.28)	0.945	1.00 (0.75–1.33)	0.997
2	1.07 (0.81–1.41)	0.641	1.02 (0.77–1.35)	0.889	0.96 (0.73–1.28)	0.798	0.91 (0.66–1.25)	0.552
3	1.41 (1.04–1.92)	**0.028**	1.38 (1.02–1.87)	**0.040**	1.42 (1.05–1.94)	**0.025**	1.47 (1.05–2.06)	**0.023**
4 or more	1.11 (0.84–1.47)	0.459	1.00 (0.75–1.32)	0.991	1.03 (0.78–1.37)	0.826	0.97 (0.71–1.33)	0.854
***Covariates*** ^***b***^								
Age, decades	1.01 (1.01–1.02)	<0.001*	0.98 (0.97–0.99)	<0.001*	0.98 (0.97–0.99)	<0.001*	0.99 (0.98–0.99)	<0.001*
Gender (Male)	0.68 (0.56–0.82)	<0.001*	0.41 (0.34–0.50)	<0.001*	0.41 (0.34–0.50)	<0.001*	0.37 (0.30–0.45)	<0.001*
Ethnicity (White)	0.53 (0.36–0.80)	0.002*	0.45 (0.30–0.67)	<0.001*	0.45 (0.30–0.68)	<0.001*	0.44 (0.27–0.73)	0.001*
Educational status (Post age 16)	0.56 (0.44–0.71)	<0.001*	0.81 (0.64–1.03)	0.092	0.75 (0.59–0.95)	**0.019**	0.88 (0.68–1.16)	0.387
Employment status (Employed)	0.86 (0.72–1.02)	0.091	0.85 (0.72–1.02)	0.072	0.89 (0.75–1.07)	0.207	0.90 (0.74–1.09)	0.270
BMI (Underweight/normal)								
Overweight	1.10 (0.90–1.34)	0.352	1.29 (1.06–1.57)	0.012*	1.28 (1.05–1.56)	**0.017**	1.33 (1.07–1.65)	0.011*
Obese	0.66 (0.52–0.83)	<0.001*	1.06 (0.84–1.33)	0.628	1.01 (0.81–1.28)	0.908	1.18 (0.92–1.52)	0.202
Other	0.79 (0.56–1.10)	0.165	0.95 (0.67–1.34)	0.758	0.88 (0.62–1.25)	0.475	0.80 (0.53–1.19)	0.270
Smoking status (Non-smoker)								
Ex-smoker	1.21 (0.99–1.47)	0.065	1.39 (1.14–1.70)	0.001*	1.37 (1.12–1.67)	0.002*	1.65 (1.33–2.05)	<0.001*
Current smoker	0.81 (0.63–1.04)	0.092	1.15 (0.90–1.49)	0.266	1.18 (0.92–1.52)	0.194	1.55 (1.18–2.04)	0.002*
Pseudo R^2^ (Nagelkerke)	0.059		0.097		0.101		0.108	

^a^Odds ratio adjusted for age, gender, ethnicity, educational status, employment status, BMI and smoking status.

^b^Odds ratio of sociodemographic factors predicting alcohol consumption independent of MSA.

MVPA = moderate to vigorous physical activity; MSA = muscle strengthening activity; HED = Heavy episodic drinking; OR = Odds ratio; CI = confidence interval.

Bold figures indicate significant p values (p<0.05); *Significant p-values following Benjamini-Hochberg correction (p<0.05).

When we adjusted for socio-demographic factors, the null association between total MVPA and alcohol consumption measures was maintained. In addition, average daily sedentary time was no longer associated with alcohol quantity [B = 0.010; OR (95% CI) = 1.01 (0.99–1.04)], frequency of HED [B = 0.009; OR (95% CI) = 1.01 (0.98–1.04)] and risky drinking status [B = 0.014; OR (95% CI) = 1.01 (0.99–1.04)]. MSA 3 days/week compared with no MSA was associated with higher odds of being in a higher category of alcohol frequency, alcohol quantity, frequency of HED and risky drinking status by 41%, 38%, 42% and 47% respectively. However, MSA 4 or more days/week compared to no MSA was not associated with higher odds of being in a higher category of alcohol frequency [B = 0.106; OR (95% CI) = 1.11 (0.84–1.47)], alcohol quantity [B = -0.002, OR (95% CI) = 1.00 (0.75–1.32)], frequency of HED [B = 0.032; OR (95% CI) = 1.03 (0.78–1.37)] or risky drinking status [B = -0.030; OR (95% CI) = 0.97 (0.71–1.33)]. Age, gender, ethnicity, educational status, BMI and smoking status were significantly associated with alcohol consumption variables independent of MSA (Table 2). Alcohol quantity per single occasion, frequent HED and possible dependence were associated with higher odds of a decrease in age by ten years. Being male and white was consistently significantly associated with alcohol consumption. Apart from drinking frequency, being overweight compared to underweight/normal was associated with higher odds of alcohol consumption. Obese individuals compared with those underweight/normal were more likely to drink frequently. Current smokers compared with non-smokers were more likely to be possibly dependent but less likely to drink frequently, drink more per occasion or engage in frequent HED. Except drinking frequency, being an ex-smoker was associated with other dimensions of alcohol consumption. There were no significant interactions between PA variables and socio-demographic factors.

Lastly, adjusting for rating of physical health diminished the association of MSA with frequency of alcohol consumption and quantity of alcohol but not frequency of HED [B = 0.0321; OR (95% CI) = 1.38 (1.01–1.88); p = 0.043] and risky drinking status [B = 0.360; OR (95% CI) = 1.43 (1.02–2.01); p = 0.036].

### Sensitivity analysis

Weighted analysis. Data was weighted to match the UK population in sensitivity analysis. In adjusted models (Table 3), the estimated odds ratio [B = -0.025; OR (95% CI) = 0.98 (0.95–1.00)] indicated that total MVPA was negatively associated with risky drinking status. This suggests that possibly dependent individuals were less likely to engage in weekly MVPA. Average daily sedentary time was not associated with any alcohol consumption variables (Table 3). However, the results changed with MSA and alcohol consumption. We observed that compared with no MSA /week, MSA 1 day/week was positively associated with alcohol quantity per occasion and frequency of HED by 40% and 47% respectively. Engaging in MSA 1 day/week and 2 days/week compared with no MSA was also significantly associated with alcohol frequency by 39% and 56% respectively. There was no observed association between MSA 3 days/week or MSA 4 days/week (compared with no MSA) with any alcohol consumption variables (Table 3). Being male [B = -0.649; OR (95% CI) = 0.52 (0.43–0.64); p<0.001], white [B = -0.620; OR (95% CI) = 0.54 (0.39–0.73); p<0.001], attaining post- age 16 education [B = -0.265; OR (95% CI) = 0.77 (0.62–0.95); p = 0.013] and being overweight [B = 0.364; OR (95% CI) = 1.44 (1.15–1.81); p = 0.002] (compared with underweight/normal) were significantly associated with frequency of alcohol consumption independent of MSA. An increase in age by ten years was associated with an increase in the odds of being in a higher category of alcohol quantity, frequent HED and risky drinking status but not alcohol frequency [B = 0.001; OR (95% CI) = 1.00 (0.99–1.01)]. Being male [B = -0.774; OR (95% CI) = 0.46 (0.38–0.56); p<0.001], white [B = -0.643; OR (95% CI) = 0.53 (0.38–0.72); p<0.001] and overweight compared with underweight/normal [B = 0.466; OR (95% CI) = 1.59 (1.27–2.01)] was associated with an increase in the odds of being on a higher category of drinking more alcohol per occasion. In terms of smoking status, we observed that the odds of being on a higher category of risky drinking status was significantly associated with being an ex-smoker [B = 0.400; OR (95% CI) = 1.49 (1.16–1.93)] or current smoker [B = 0.358; OR (95% CI) = 1.43 (1.09–1.88)].

**Table 3:** Association between physical activity and alcohol consumption (weighted).

Independent variables	Frequency of alcohol consumption		Quantity of alcohol consumed (units)		Frequency of HED		Risky drinking status	
Weighted N = 1703	OR (95% CI)	P	OR (95% CI)	P	OR (95% CI)	P	OR (95% CI)	P
***Unadjusted models***								
Total MVPA, hrs*/week*	1.01 (1.00–1.03)	0.156	1.00 (0.99–1.02)	0.863	1.01 (0.99–1.03)	0.404	0.98 (0.96–1.00)	0.051
Average daily sedentary time, *hrs/day*	1.01 (0.99–1.04)	0.278	1.04 (1.01–1.06)	0.004*	1.05 (1.03–1.08)	<0.001*	1.04 (1.01–1.07)	0.006*
Frequency of MSA, *days/week* (0 days/week as reference)								
1	1.31 (0.97–1.77)	0.080	1.40 (1.04–1.88)	**0.029**	1.43 (1.06–1.93)	0.020*	1.25 (0.90–1.72)	0.182
2	1.48 (1.02–2.15)	**0.041**	1.00 (0.69–1.45)	0.999	1.01 (0.70–1.47)	0.942	0.89 (0.59–1.36)	0.595
3	1.10 (0.77–1.57)	0.599	1.30 (0.91–1.85)	0.150	1.52 (1.06–2.16)	0.022*	1.40 (0.96–2.05)	0.083
4 or more	1.20 (0.87–1.64)	0.266	1.29 (0.95–1.77)	0.107	1.21 (0.88–1.65)	0.245	1.03 (0.73–1.46)	0.869
***Adjusted models*** ^***a***^								
Total MVPA, *hrs/week*	1.01 (0.99–1.02)	0.584	0.99 (0.98–1.01)	0.501	1.00 (0.98–1.02)	0.980	0.98 (0.95–1.00)	**0.030**
Average daily sedentary time, *hrs/day*	1.00 (0.98–1.03)	0.814	1.01 (0.98–1.03)	0.703	1.01 (0.99–1.04)	0.342	1.01 (0.98–1.04)	0.541
Frequency of MSA, *days/week* (0 days/week as reference)								
1	1.39 (1.02–1.89)	**0.039**	1.47 (1.08–2.00)	**0.016**	1.40 (1.02–1.91)	**0.035**	1.27 (0.90–1.79)	0.167
2	1.56 (1.06–2.29)	**0.025**	1.10 (0.75–1.61)	0.639	1.09 (0.74–1.60)	0.665	1.00 (0.64–1.56)	0.993
3	1.09 (0.75–1.57)	0.655	1.26 (0.88–1.83)	0.213	1.34 (0.93–1.94)	0.122	1.25 (0.83–1.88)	0.281
4 or more	0.92 (0.66–1.28)	0.614	1.09 (0.78–1.51)	0.621	0.91 (0.66–1.27)	0.585	0.83 (0.57–1.19)	0.309
Pseudo R^2^ (Nagelkerke)	0.079		0.104		0.113		0.109	

^a^Odds ratio adjusted for age, gender, ethnicity, educational status, employment status, BMI and smoking status.

MVPA = moderate to vigorous physical activity; MSA = muscle strengthening activity; HED = Heavy episodic drinking; OR = Odds ratio; CI = confidence interval.

Bold figures indicate significant p values (p<0.05); *Significant p-values after Benjamini-Hochberg correction (p<0.05).

**Fig 1:**
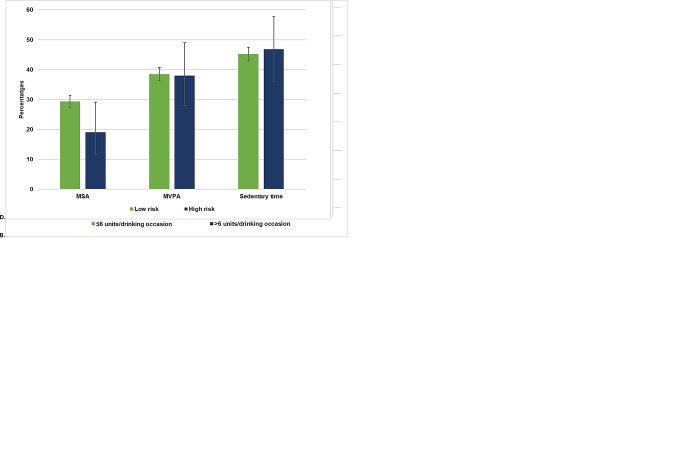
(A–D) Percentage of participants meeting PA guidelines and risky sedentary behaviour by alcohol consumption. (A) Frequency of alcohol consumption (B) Quantity of alcohol consumed (units/week) (C) Frequency of heavy episodic drinking (D) Risky drinking status.

Binary logistic regression models. The percentage of participants meeting UK PA guidelines (MVPA and MSA) and engaging in risky sedentary behaviour by dichotomized alcohol consumption measures is illustrated in Fig 1A–1D below. The cluster bar charts demonstrated that participants who drank alcohol more frequently met MVPA and MSA guidelines but also spent slightly more time in sedentary behaviour (≥7.5 hrs/day) compared with current abstainers (Fig 1A). In unadjusted analysis, frequent drinking (versus being a current abstainer) was associated with a higher likelihood of meeting MVPA guidelines [B = 0.326; OR (95% CI) = 1.39 (1.05–1.82)] but this association became non-significant after we controlled for covariates [B = -0.222; OR (95% CI) = 1.25 (0.94–1.66)]. Drinking more than 6 units/drinking occasion was marginally associated with meeting MVPA guidelines but those who were less likely to meet the MSA guidelines were more likely to drink ≤6 units/drinking occasion and spent more time in sedentary behaviour compared with those drinking ≤6 units/drinking occasion (Fig 1B). In adjusted models, drinking more than 6 units/drinking occasion was not significantly associated with meeting MVPA [B = 0.225; OR (95% CI) = 1.25 (0.93–1.69)] or MSA [B = -0.173; OR (95% CI) = 0.84 (0.61–1.16)] guidelines.

A large proportion of participants who engaged in frequent HED also spent the most time in sedentary behaviour (Fig 1C). There was minimal difference in terms of meeting the MVPA and MSA guidelines for participants who engaged in no HED compared those engaged in frequent HED. However, and risky sedentary time was significantly positively associated with frequent HED in both unadjusted [B = 0.485; OR (95% CI) = 1.62 (1.35–1.96)] and adjusted models [B = 0.236; OR (95% CI) = 1.27 (1.04–1.55)]. There was marginal difference in terms of MVPA between high-risk and low-risk drinkers, but high-risk drinkers were more likely to be sedentary (Fig 1D). High risk drinkers were also less likely to meet MSA guidelines in both adjusted [B = -0.531; OR (95% CI) = 0.59 (0.32–1.07)] and unadjusted models [B = -0.576; OR (95% CI) = 0.56 (0.32–1.00)].

## Discussion

This study contributes to our understanding of the association between PA and alcohol consumption among adults during the COVID-19 pandemic. The main findings show that amongst a self-selected sample of participants, adults who engage in MSA are more likely to report drinking frequently, drinking more per drinking occasion, frequent HED and be at risk of alcohol dependence. We observed that being young, male, and white was significantly associated with alcohol consumption independent of MSA. Smoking status and BMI were also associated with higher odds of alcohol consumption. Total MVPA and average daily sedentary time were not associated with alcohol consumption.

Despite the small effect sizes observed, the finding that MSA rather than MVPA is associated with alcohol consumption is interesting and contradicts the current evidence. There is no known explanation for the association we observed between MSA and alcohol consumption in the literature. However, COVID-19 related lockdown measures may explain the novelty of our findings. The choice of MSA over MVPA can be related to accessibility since adults were allowed only one bout of exercise outside while gyms were closed. Muscle strengthening activities such as Pilates, yoga and body weight exercises may have been easier to perform at home during lockdown restrictions than aerobic activities such as walking, cycling and running. Because drinking is deeply embedded in society and culture, it may not be viewed as an important health risk behaviour compared with others such as smoking or illegal drug use [32]. One might expect that individuals engaging in MSA frequently are health conscious and might make better health choices including limiting alcohol consumption. However, these individuals may have used drinking to counteract negative psychological consequences of lockdown restrictions such as stress, boredom or frustration. Further, existing research on compensatory health behaviours suggests that individuals may mitigate the negative effects of less healthy choices by engaging in more health promoting activities [33]. For instance, having exercised in the morning, one may justify excessive drinking by rationalising that they have earned it. In women, exercise as a compensatory strategy for drinking has been associated with greater body dissatisfaction and weight concerns [34]. Our findings support this contention as being overweight or obese was associated with alcohol consumption independent of MSA. Nonetheless, this does not explain why MSA 4 or more days/week compared with no MSA was not associated with higher odds of being on a higher category of alcohol consumption. We speculate that individuals who engage in MSA at this level may be professional athletes or more health-conscious individuals who do not use exercise to compensate for drinking.

Several unknown factors and mechanisms may play a role in the link between MSA and alcohol consumption during the COVID-19 pandemic. When considered simultaneously in adjusted models, socio-demographic factors strengthened the observed association between MSA and alcohol consumption compared with the unadjusted model. This suggests that socio-demographic confounders measured here cannot fully explain the association between MSA and alcohol consumption. As an example, sports participation or sports club membership may be a possible moderator of this association [12]. Individual differences in the COVID-19 experiences such as acquiring the virus and its long-term effects can influence health behaviours and their interactions [35]. Although the evidence suggests that sedentary behaviour and alcohol consumption increased during the COVID-19 pandemic [36,37], we did not observe a significant association when socio-demographic confounders were considered.

These exploratory findings add to the body of knowledge regarding the association of alcohol consumption with PA [9,10,14] and sedentary behaviour [11]. Previous cross-sectional studies have mainly examined MVPA and its association with alcohol consumption. We did not observe that individuals who engaged in high levels of MVPA were more likely to consume higher levels of alcohol consumption as other studies have found [13,38,39]. Few studies have reported no association between MVPA and alcohol consumption. A survey of the Austrian adult population using the IPAQ, a more accurate measure of self-reported PA and frequency-quantity measures of alcohol consumption found similar results to the present study. The authors found that PA was not associated with a higher probability of alcohol consumption [40]. A similar study on Austrian college students also did not observe a positive association between PA and alcohol consumption [41]. Considering much of the evidence pointing to a positive association has been conducted in the USA, it is likely that cross-national differences in alcohol consumption and PA may contribute to conflicting findings. In addition, different assessment methods of PA and alcohol consumption may contribute to varying findings. The most significant difference, however, is the COVID-19 pandemic context in this study as lockdown restrictions may have limited opportunities for aerobic activity.

There are several strengths to this study. The sample was relatively large, and data included a wide range of covariates. Alcohol consumption was operationalised using the AUDIT-C questionnaire, a valid and reliable instrument [26]. Moreover, we differentiated PA in terms of sedentary behaviour, MVPA and frequency of MSA which provides a more nuanced understanding of the association between PA and alcohol consumption. The following limitations must be considered when interpreting the findings. Firstly, participants were self-selected and largely older, female, and White which limits the generalisability of results. Further evidence is required to examine whether our findings hold among minority ethnic and less socially advantaged groups. As with previous studies, physical activity and alcohol consumption was self-reported which might introduce recall or social desirability bias. Using objective measures of PA (accelerometers) may provide more reliable findings. Although frequency of MSA is predominantly used in public health research, it does not provide the nuanced insight required to establish optimal doses in future interventions [42]. To further explore this link between MSA and alcohol consumption, future studies must consider duration, intensity, and type of activity (e.g., bodyweight vs weight machines or equipment and single vs multi-joint activities) to provide more nuanced insight of this association. Future interventions and policies must take into consideration how different constructs of PA and drinking might fit into an individual’s lifestyle to provide the necessary support. The findings cannot infer causality due to the cross-sectional design of the study. However, the cross-sectional design holds value in exploring the nature of this association in the UK population. Longitudinal analysis of the temporal relationship between MSA and alcohol consumption can be considered in future research.

## Conclusion

PA and alcohol consumption are complex behaviours and have significant impacts on health. The COVID-19 pandemic and its consequent control measures in the UK impacted adults’ PA and drinking. During COVID-19, MSA but not MVPA was associated with increased alcohol consumption. As outdoor activity was restricted during lockdown, MSA in the form of home-based activities may have been used to counteract boredom and frustration. This finding is useful for identifying and targeting groups who drink at risky levels for future research and public health policy regarding future pandemics. Such public health policies may involve raising awareness through messaging about possible health harms associated with negative behaviours such as risky drinking whilst promoting positive behaviours such as physical activity. Finally, although there is consistent evidence of the association between MVPA and alcohol consumption, holistic approaches of measuring PA that include MSA should be considered.
